# Effects of Caffeine Ingestion on Anaerobic Capacity in a Single Supramaximal Cycling Test

**DOI:** 10.3389/fnut.2018.00086

**Published:** 2018-09-20

**Authors:** Willian E. Miyagi, Romulo C. Bertuzzi, Fabio Y. Nakamura, Rodrigo A. B. de Poli, Alessandro M. Zagatto

**Affiliations:** ^1^Laboratory of Physiology and Sport Performance (LAFIDE), Bauru, Brazil; ^2^Post-Graduate Program in Movement Sciences, Sao Paulo State University–UNESP, School of Science, Bauru, Brazil; ^3^School of Physical Education and Sport, University of São Paulo, São Paulo, Brazil; ^4^Department of Medicine and Aging Sciences, “G. d'Annunzio” University of Chieti-Pescara, Pescara, Italy

**Keywords:** caffeine, anaerobic capacity, performance, running, ergogenic aids

## Abstract

The aim of this study was to verify the effects of caffeine on anaerobic capacity estimated by the sum of the estimated glycolytic [E_[La]_] and phosphagen [E_PCr_] metabolism based on blood lactate and excess post-oxygen consumption responses (AC_[La−]+EPOCfast_). Fourteen male cyclists were submitted to a graded exercise test to determine the maximal oxygen uptake ($\overset{{}^{\circ}}{V}O_{2max}$) and intensity associated with$\overset{{}^{\circ}}{\;V}O_{2max}$ (i$\overset{{}^{\circ}}{V}O_{2max}$). Subsequently, the participants performed two supramaximal efforts at 115% of i$\overset{{}^{\circ}}{V}O_{2max}$ to determine the AC_[La−]+EPOCfast_, after previous supplementation with caffeine (6 mg·kg^−1^) or a placebo (dextrose), in a cross over, randomized, double blind, and placebo-controlled design. The time to exhaustion was higher in the caffeine (186.6 ± 29.8 s) than in the placebo condition (173.3 ± 25.3 s) (p = 0.006) and a significant correlation was found between them (r = 0.86; P = 0.00008). Significant differences were not found between AC_[La−]+EPOCfast_ values from the placebo (4.06 ± 0.83 L and 55.2 ± 5.7 mL·kg^−1^) and caffeine condition (4.00 ± 0.76 L and 54.6 ± 5.4 mL·kg^−1^); however, a significant correlation was observed only for AC_[La−]+EPOCfast_ expressed in absolute values (r = 0.74; p < 0.002). The E_[La]_ and E_PCr_ also presented no significant differences and they were significantly correlated (r = 0.82 and r = 0.55, respectively; p < 0.05). We conclude based on the overall comparison of mean values between two treatments that acute caffeine ingestion improves the time to exhaustion but does not affect anaerobic capacity estimation.

## Introduction

Acute caffeine ingestion has been shown to cause an increase in the excessive post-exercise oxygen consumption (EPOC) after resistance training (1), time to exhaustion during supramaximal effort (2) and other performance parameters. Moreover, the improvement in time to exhaustion during a supramaximal effort after caffeine intake caused by changes in oxygen consumption ($\overset{{}^{\circ}}{V}O_{2}$)at the exhaustion moment, possibly affecting the phosphagen energy system estimation (2). In addition, some studies have shown that acute ingestion of caffeine increased time to exhaustion during a supramaximal test and anaerobic capacity estimated by the maximal accumulated oxygen deficit (MAOD) (3–5). Bell et al. (3) found a consequent increase in peak lactate concentration after a supramaximal effort to determine MAOD with caffeine supplementation. However, other studies have not shown significant effects of caffeine ingestion on exercise blood lactate concentration after supramaximal efforts to determine MAOD (4, 5). The possible changes in lactate concentrations could cause changes in the estimation of glycolytic system and consequently alter the estimation of capacity anaerobic when estimated by blood lactate concentration and EPOC responses (6–8), and consequently can alters the anaerobic capacity.

MAOD is one of the most accepted methods for evaluating an individual's maximal capacity of ATP resynthesis by means of the non-mitochondrial metabolism, which is determined by means of calculations involving $\overset{{}^{\circ}}{V}O_{2}$ measurements during several submaximal exercise sessions and one supramaximal exhaustive exercise session (9). However, the time-consuming nature of MAOD determination can discourage its use in routine athlete training and, for this reason, the anaerobic capacity has been alternatively estimated using only a single supramaximal effort (AC_[La−]+EPOCfast_) (6–8). AC_[La−]+EPOCfast_ determination is based on an estimation of the oxygen equivalent from the glycolytic (E_[La−]_) and phosphagen (E_PCr_) pathways, considering the accumulated blood lactate (10) and the fast component of the excessive post-exercise oxygen consumption (EPOC_fast_) (11), respectively. The AC_[La−]+EPOCfast_ is an advantageous method for anaerobic capacity assessment and its validity has been demonstrated by the absence of significant differences with the conventional MAOD determined in cycling (6, 7), running (8), and table tennis (12, 13). Furthermore, AC_[La−]+EPOCfast_ method has shown high reliability (intraclass correlation coefficient [ICC] = 0.87 and typical error = 0.27 L) and significant associations with mechanical variables assessed in a 30 s maximal effort (14), besides to be sensitive in distinguishing individuals with different physical conditioning status (15).

The E_[La−]_ is estimated by the values of blood lactate elevation from baseline during exercise, which could be influenced by factors related to the efflux of this metabolite from the muscle (16). On the other hand, the E_PCr_ is estimated by computing the EPOC_fast_, which could be affected by any factor that causes alterations in the parameters related to this metabolic pathway calculation (e.g., oxygen uptake attained at exhaustion) (17). Thus, any intervention capable of modifying these responses (i.e., blood lactate and post-exercise oxygen consumption) could compromise the reliability of the AC_[La−]+EPOCfast_. For example, the AC_[La−]+EPOCfast_ was increased after acute supplementation with sodium bicarbonate due to E_[La]_ alteration (16). However, it is not yet clear whether AC_[La−]+EPOCfast_ is sensitive to possible changes caused in the EPOC_fast_, induced for example by caffeine ingestion (1, 2).

De Poli et al. (2) found no effect of caffeine on AC_[La−]+EPOCfast_ values determined in running, but suggested possible alterations in the relative contributions of the glycolytic (+9.3% with ~68% possibly positive effect) and phosphagen metabolic (−5.4% with ~76% possibly negative effect) pathways. Nevertheless, anaerobic evaluations are widely used in cycling (18) and investigations into the effects of caffeine on cycling AC_[La−]+EPOCfast_ are missing. Importantly, different physiological responses can be observed in cycling compared to running (19) and MAOD seems to be affected by the exercise mode (20). Thus, considering the possible “fluctuations” in determining AC_[La−]+EPOCfast_ that could be caused by these changes in E_PCr_ and E_[La]_, it is necessary to investigate the effects of acute caffeine intake on the estimation of “anaerobic” capacity using this protocol in cycling.

Therefore, the aim of the present study was to verify the effects of acute caffeine ingestion on “anaerobic” capacity estimated by the AC_[La−]+EPOCfast_ in cycling. We hypothesized that caffeine intake would improve performance in the supramaximal effort and change the relative energetic contribution of E_[La−]_ and E_PCr_ on AC_[La−]+EPOCfast_ method, as observed in running (2).

## Materials and methods

### Subjects

Fifteen male mountain bikers were considered eligible to participate in the study. Participants were recruited from regional cycling groups. To be included, they should be healthy, without any vascular disease, metabolic disorders, recent muscle-skeletal, or joint injuries and should not have used nutritional supplements as beta-alanine and creatine or pharmacological substances for at least 3 months. One participant was excluded from the study due to the inclusion criteria and therefore the final sample size was composed of 14 bikers. Five subjects had been competing at the regional level for at least 10 years. The other nine subjects reported at least 1 year of regular training and competition experience. The average weekly training volume reported by these individuals was 203 ± 122 km per week with a training frequency of 3–6 times.

One individual was excluded from the study due to high self-reported habitual daily caffeine ingestion (~780 mg·day^−1^). The participants' daily intake of caffeine was estimated for 3 days prior to the commencement of the study and reported to be 53.4 ± 39.8 mg·day^−1^. All subjects were prohibited to consume any food or drink containing caffeine (i.e., tea, coffee, soft drinks, energy drinks, chocolate, and others) and alcohol, as well as were instructed to refrain to perform vigorous physical activity for at least 24 h before each test session. In addition, all subjects were instructed to consume their habitual meal. The characteristics of the subjects are presented in Table 1.

**Table 1:** Characteristics of the subjects (n = 14).

	**Age (years)**	**Height (cm)**	**Body mass (kg)**	Lean mass*(kg)	LM-LL*(kg)	Fat*(%)
Mean ± SD	30 ± 6	179.4 ± 8.4	73.2 ± 11.4	58.3 ± 8.5	20.9 ± 3.4	15.4 ± 4.9

Values are mean ± SD. LM-LL, Lean mass of lower limbs. ^*^measured by DXA (Discovery, Hologic, USA).

The subjects were informed about the risks and benefits of the procedures and signed a written consent prior to commencing study participation. All procedures were approved by the local Ethics Committee (Protocol 645 784/2014) and were conducted in accordance with the Helsinki Declaration.

### Experimental design

**Figure 1:**
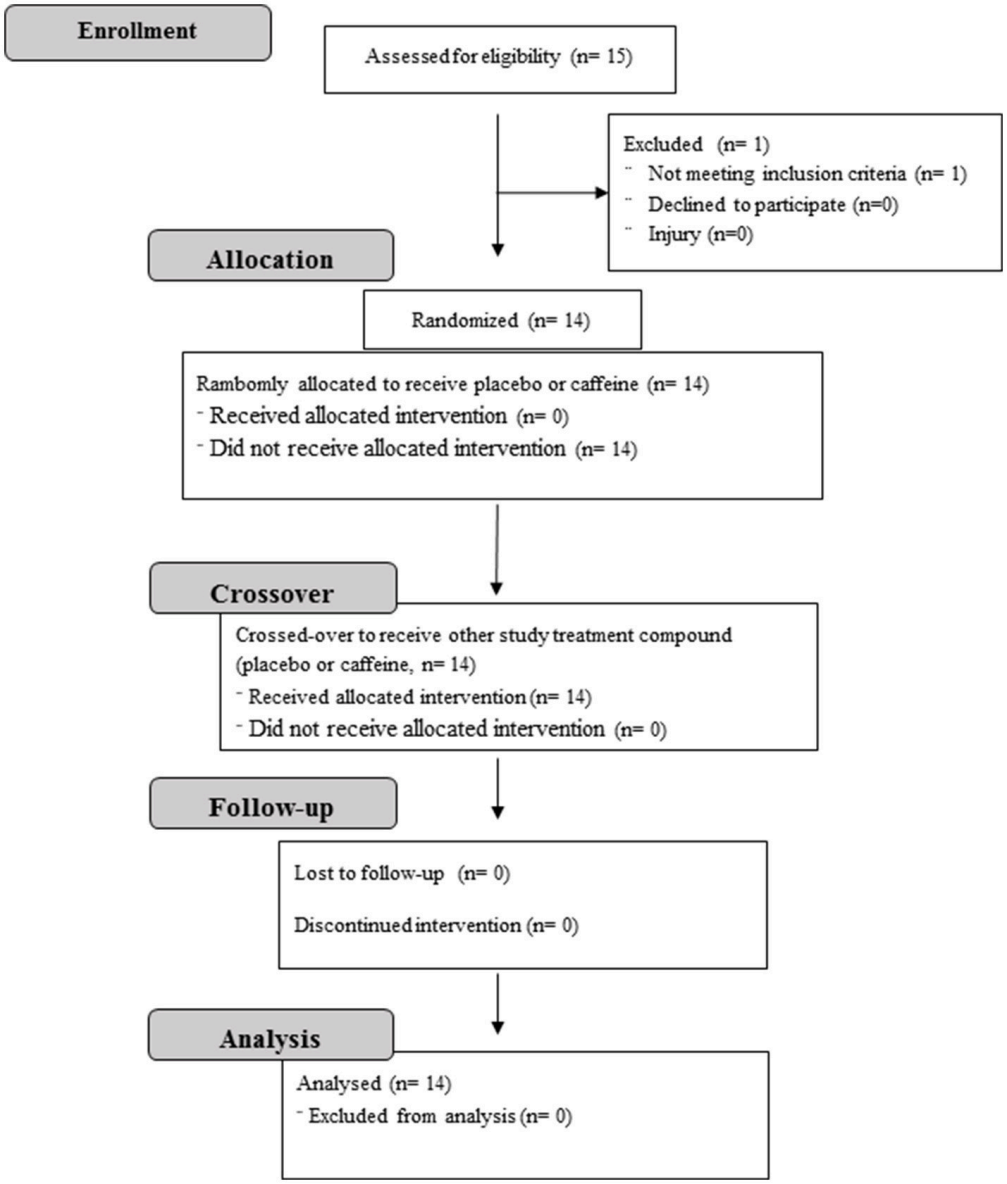
Flow diagram of the study design.

The study design was a placebo-controlled double-blind crossover randomized trial. Figure 1 presents a flow diagram of the study. Initially, the subjects were submitted to a graded exercise test (GXT) to determine the maximal oxygen uptake ($\overset{{}^{\circ}}{V}O_{2max}$) and intensity associated with $\overset{{}^{\circ}}{V}O_{2max}$ (i$\overset{{}^{\circ}}{V}O_{2max}$). Next, they performed two supramaximal efforts at 115% of i$\overset{{}^{\circ}}{V}O_{2max}$ to determine the AC_[La−]+EPOCfast_, with or without (placebo condition) caffeine supplementation. The three sessions were separated by a minimum of 48 h. In all tests, the warm-up was standardized at 100 W for 5-min and was carried out 5-min before the tests.

All exercise tests were performed on an electromagnetic cycle ergometer (Lode-Excalibur, Lode, Netherlands). The subjects were instructed to adopt a preferred cadence between 70 and 90 rpm and to maintain the chosen cadence with a maximum variation of ± 5 rpm throughout the tests. The procedures for each study session were applied in an environment with controlled temperature and humidity (20 ± 1°C and 61 ± 8%, respectively).

For all laboratory visits, the subjects were informed to maintain the normal diet during the day and to make a meal between 2 and 4 h before to start the exercise procedures.

### Physiological and metabolic data collection

The respiratory responses were measured breath-by-breath by a stationary gas analyzer (Quark CPET, COSMED, Rome, Italy). The gas analyzer was calibrated before each test session using gas samples with known concentrations (5.00% CO_2_ and 16.02% O_2_, White Martins®, Osasco, Brazil) and room air, while the turbine was calibrated through a 3-L syringe (Hans-Rudolf, USA). The $\overset{{}^{\circ}}{V}O_{2}$ obtained during the tests was smoothed each 5 points and interpolated at 1 s intervals through OriginPro 9.0 software (OriginLab Corporation, Microcal, Massachusetts, USA). The heart rate (HR) was measured by a transmitter belt with wireless connection to the gas analyzer (Wireless HR 138 Monitor, COSMED, Rome, Italy),while the rating of perceived exertion (RPE) was assessed using the 6–20 Borg scale (21).

The blood lactate concentration ([La^−^]) was measured from blood samples collected from the ear lobe (25 μl) at rest ([La^−^]_Rest_) (prior to warm-up) and 3, 5, and 7 min after each maximal test to determine the peak lactate concentration ([La^−^]_Peak_). The blood samples were collected and stored in *Eppendorf* tubes containing 50 μL of 1% sodium fluoride and then analyzed using a YSI 2300 STAT *(Yellow Spring Instruments, Ohio, USA)* (typical error of ± 2%).

The subjects remained seated for 10 min to determine the $\overset{{}^{\circ}}{V}O_{2}$ baseline and [La^−^]_Rest_. Baseline $\overset{{}^{\circ}}{V}O_{2\;}$was considered as the mean of the final 2 min, while the exhaustion $\overset{{}^{\circ}}{V}O_{2}$ was considered the mean of the final 30 s of the supramaximal test.

### Body composition analysis

Body composition was measured by dual-energy X-ray absorptiometry (DXA) using the Discovery corporal scanner (Hologic, Sunnyvale, USA). The body segmentation analysis was carried out with the horizontal line positioned above the bowl slightly above iliac crest. The angular lines that define the pelvic triangle were sectioned at the femur, and the vertical line positioned between the legs dividing the two feet. The lean mass of the lower limbs (LM-LL) was considered the sum of the right and left legs, not considering the bone mass values (7).

### Graded exercise test (GXT)

The GXT was designed to induce exhaustion in ~8–12 min (22). The initial power output was set at 100–150 W with increments of 25 W every 2 min until voluntary exhaustion or the inability to maintain the pre-defined cadence (23). In each test stage, $\overset{{}^{\circ}}{V}O_{2}$ measured during the final 30 s was averaged. The highest 30 s average $\overset{{}^{\circ}}{V}O_{2}$ obtained during the test was considered as the $\overset{{}^{\circ}}{V}O_{2max}$, considering the verification of a plateau in $\overset{{}^{\circ}}{V}O_{2\;}$(variation in $\overset{{}^{\circ}}{V}O_{2}$ < 2.1 mL·kg^−1^·min^−1^ between the final and penultimate stage of exercise). Secondary criteria were: maximal HR ≥ 90% of predicted maximal value, respiratory exchange ratio (RER) ≥ 1.10, and peak lactate ≥ 8.0 mmol·L^−1^ (22). The i$\overset{{}^{\circ}}{V}O_{2max}$ was assumed as the lowest intensity at which the $\overset{{}^{\circ}}{V}O_{2max}$ was attained (24).

### Caffeine supplementation and supramaximal efforts

The subjects ingested 6 mg·kg^−1^ of caffeine or a placebo (dextrose) (Neonutri, Minas Gerais, Brazil) 1 h before each supramaximal effort (Gemini Pharmaceutical Ingredients Industry Ltda, Anápolis, GO, Brazil), in a double blind and randomized fashion. The caffeine and placebo were contained in identical gel capsules, which were produced in our laboratory using a manual capsule filling machine. The caffeine dosage used was chosen as it has been proved to cause changes in the excessive post-exercise oxygen consumption (1).

The individuals performed the supramaximal efforts at 115% of i$\overset{{}^{\circ}}{V}O_{2max}$ to determine the time to exhaustion at this intensity and the corresponding AC_[La−]+EPOCfast._. The E_PCr_ was estimated by the EPOC_FAST_, analyzed using a bi-exponential model (Equation 1) (Origin PRO 9.0, OriginLab Corporation, Micrical, Massachusetts, USA) and corresponded to the product between A_1_ and τ_1_ (Equation 2) (6–8). The E_[La]_ was estimated by the difference between the [La^−^]_Peak_ and [La^−^]_Rest_, considering each 1.0 mmol·L^−1^ of accumulated lactate equivalent to 3 mL·kg^−1^ of oxygen (10). The AC_[La−]+EPOCfast_ was determined from the sum of oxygen equivalents of E_[La−]_ and E_PCr_ pathways (Equation 3)

(1) $$\dot{\text{V}}{\text{O}}_{2\left(t\right)}=\;\dot{\text{V}}{\text{O}}_{2\left(\text{Rest}\right)}+\;A_{1}\left[e^{-\left(\frac{t}{\tau 1}\right)}\right]\text{ + }{\text{A}}_{2}\left[{\text{e}}^{\text{-}\left(\frac{\text{t}}{\tau \text{2}}\right)}\right]$$

(2) $${\text{E}}_{\text{PCr}}{\text{=A}}_{1}\tau_{\text{1}}$$

(3) $$AC_{\left[\text{La-}\right]\text{+EPOCfast}}=\;{\text{E}}_{\text{PCr}}{\text{+E}}_{\left[\text{La-}\right]}$$

Where$\overset{{}^{\circ}}{\;V}O_{2\left(t\right)}$ corresponds to the oxygen uptake at time t, $\overset{{}^{\circ}}{V}O_{Rest}\;$is the rest oxygen uptake, A_1_ is the amplitude, and τ_1_ is the constant time.

In addition, the oxidative metabolism contribution (E_OXID_) was estimated considering the accumulated $\overset{{}^{\circ}}{V}O_{2}$ during the supramaximal effort using the trapezoidal method, excluding the rest values.

#### Statistical analysis

The sample size was calculated (software G^*^Power) based on power analysis, taking into consideration the statistical power of 90%, α error probability of 0.05, and the effect size estimated from AC_[La−]+EPOCfast_ mean and standard deviation differences in placebo and caffeine supplemented conditions (2), resulting in a minimum sample size of 13 participants. The results are presented as mean ± SD and 95% confidence interval (CI95%). Initially, the data were submitted to the Shapiro-Wilk test to verify data normality. The variables related to AC_[La−]+EPOCfast_ in the caffeine and placebo conditions were compared using the paired t-test. The association was analyzed using the Pearson's product-moment correlation test. In all tests a level of significance of 5% was assumed. In addition, the data were analyzed qualitatively by magnitude-based inference and expressed as raw mean differences. The threshold values for Cohen's d statistical power were considered as >0.2 (small), >0.5 (moderate), and >0.8 (large). The chances of a possible substantial benefit or harm were calculated [assuming the value of 0.2 multiplied by the between-subject deviation as the smallest worthwhile change (SWC)].

## Results

Table 2 displays the physiological responses at exhaustion in the GXT. All subjects reached the criteria to confirm $\overset{{}^{\circ}}{V}O_{2max}$ determination.

**Table 2:** Values of heart rate (HR), respiratory exchange ratio (RER), peak blood lactate concentration ([La^−^]_Peak_), maximal oxygen uptake ($\overset{{}^{\circ}}{V}O_{2max}$), intensity associated with $\overset{{}^{\circ}}{V}O_{2max}$ (i$\overset{{}^{\circ}}{V}O_{2max}$), and total time obtained in graded exercise test (n = 14).

	**HR (bpm)**	**RER**	**[La^−^]_Peak_ (mmol·L^−1^)**	**$\overset{{}^{\circ}}{V}O_{2max}$ (mL·kg^−1^·min^−1^)**	**i$\overset{{}^{\circ}}{V}O_{2max}$ (W)**	**Total time (min)**
Mean ± SD	186.2 ± 8.3	1.21 ± 0.06	10.0 ± 1.9	52.8 ± 8.6	298.6 ± 48.4	14.5 ± 4.0
(CI95%)	(181.1 to 191.2)	(1.17 to 1.25)	(8.9 to 11.2)	(47.8 to 57.8)	(270.6 to 326.6)	(12.1 to 16.9)

**Figure 2:**
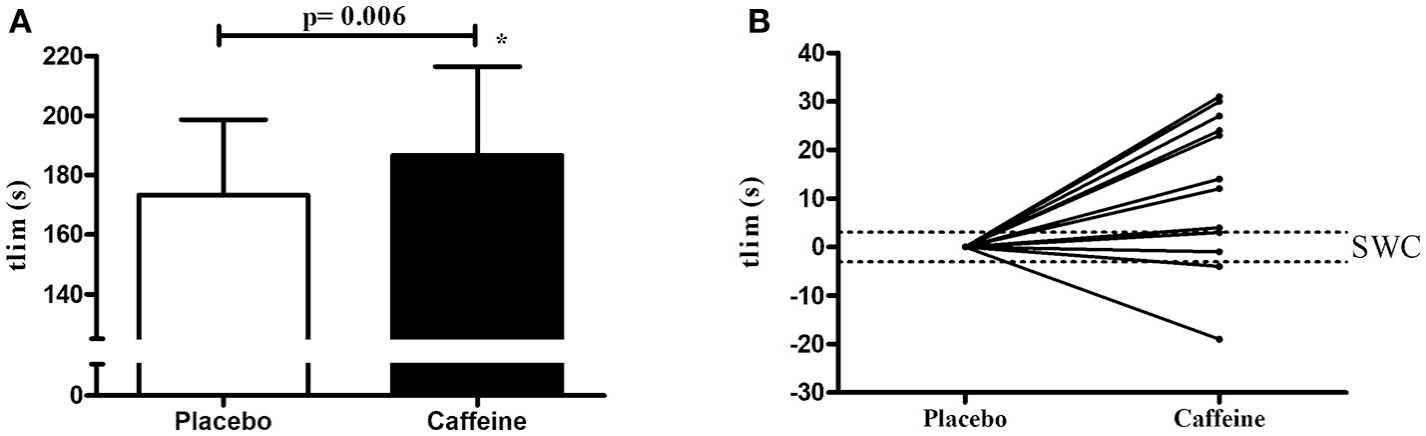
Time to exhaustion (tlim) measured during supramaximal effort at 115% of i$\overset{{}^{\circ}}{V}O_{2max}$ under placebo and caffeine conditions. **(A)** shows the values in mean and standard deviation, while the figure **(B)** shows individual changes relative to placebo values and in relation to boundaries of smallest worthwhile change (SWC).

**Figure 3:**
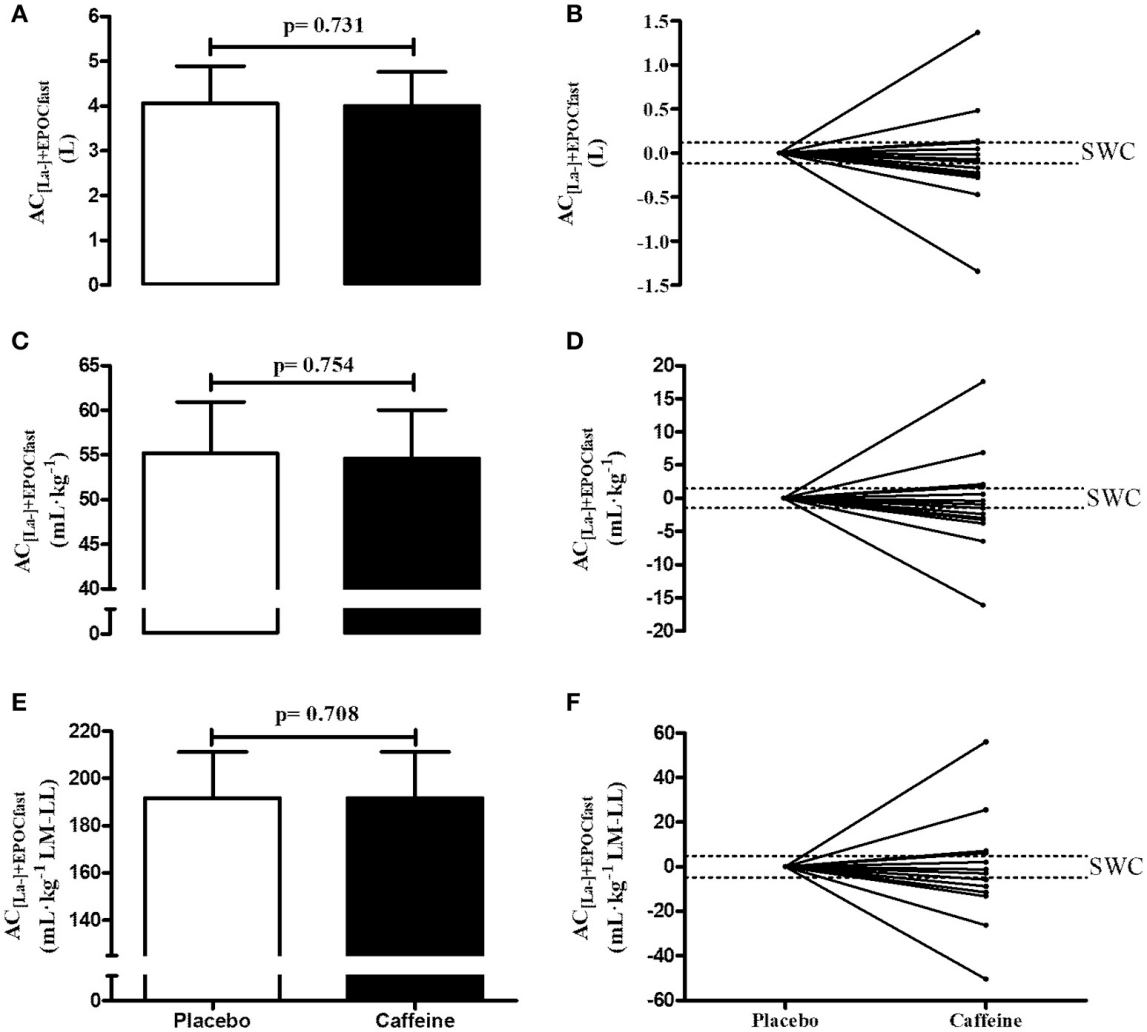
AC_[La−]+EPOCfast_ values in the caffeine and placebo conditions expressed in values absolute **(A,B)**, relative to body mass **(C,D)** and relative to lean mass of lower limbs **(E,F)** (n = 14). **(A,C,F)** show the values in mean and standard deviation, while the figures **(B,D,F)** show individual changes relative to placebo values and in relation to boundaries of smallest worthwhile change (SWC).

The 115% of i$\overset{{}^{\circ}}{V}O_{2max}$ was 335.2 ± 49.7 W (CI95% = 306.5 to 363.9 W). Figure 2 shows in mean ± SD and individual values that the time to exhaustion (tlim) was higher (Δ% = +7.8%) in the caffeine condition (CI95% = 169.4 to 203.9 s) than in the placebo condition (CI95% = 158.7 to 187.9 s) (P = 0.006) (Figure 2A), with 10 out of 14 subjects with performance higher than SWC boundary (Figure 2B). Significant correlations were found between the conditions in tlim (r = 0.86; CI95% = 0.60 to 0.95; p = 0.00008).

(Figures 3A,C,E) shows in mean ± SD the individual values of the AC_[La−]+EPOCfast_ values determined in the caffeine (CI95% = 3.56 to 4.44 L; CI95% = 51.5 to 57.7 mL·kg^−1^; CI95% = 180.3 to 202.8 mL·kg^−1^LM-LL) and placebo (CI95% = 3.57 to 4.54 L; CI95% = 51.9 to 58.5 mL·kg^−1^; CI95% = 180.3 to 202.8 mL·kg^−1^ LM-LL) conditions. There were no differences between the AC_[La−]+EPOCfast_ values expressed in absolute or relative terms (p > 0.708). In addition, when the data are analyzed individually (Figures 3B,D,F), 10 subjects from 14 participants modified the anaerobic capacity beyond upper/lower SWC limits (i.e., ~4 subjects were “positive” responders and ~7 subjects were “negative” responders), evidencing high inter-individual variability, while ~3 subjects were “non-responders.” Significant correlation was found only for the AC_[La−]+EPOCfast_ expressed in absolute values (r = 0.74 and p = 0.002), but not when expressed in relative values (r = 0.12 and p = 0.68 for relative to body mass and r = 0.40 and p = 0.16 for relative to lean mass of lower limbs).

Table 3 shows the $\overset{{}^{\circ}}{V}O_{2}$, RPE, E_OXID_, E_[La]_, and E_PCr_ variables at exhaustion during the supramaximal efforts after caffeine or placebo supplementation. The exhaustion $\overset{{}^{\circ}}{V}O_{2}$ and RPE in both conditions were not statistically different and demonstrated significant correlations. Furthermore, the E_[La]_ and E_PCr_ were not different and presented significant correlations (between conditions), while the E_OXID_ was higher in the caffeine than in the placebo condition.

**Table 3:** Values of oxygen uptake ($\overset{{}^{\circ}}{V}O_{2}$) at exhaustion, rate of perceived exertion (RPE), and energetic contribution measured at 115% of i$\overset{{}^{\circ}}{V}O_{2max}$ after caffeine and placebo supplementation (n = 14).

	**Placebo**	**Caffeine**	**p-value**	**Δ%**	**Pearson's r (CI95%)**
$\overset{{}^{\circ}}{V}O_{2}$ Exhaustion (mL·kg^−1^·min^−1^)	51.2 ± 8.7 (46.2 to 56.2)	51.3 ± 8.5 (46.4 to 56.2)	0.753	±0.4	0.98^¥^ (0.95 to 0.99)
RPE	18 ± 2 (17 to 19)	18 ± 2 (17 to 19)	1.000	-0.3	0.83^¥^ (0.53 to 0.95)
E_OXID_ (L)	7.44 ± 2.68 (5.90 to 8.99)	8.15 ± 2.96 (6.44 to 9.86)^*^	0.014	±9.7	0.95^¥^ (0.84 to 0.98)
E_[La−]_ (L)	2.53 ± 0.59 (2.19 to 2.87)	2.55 ± 0.51 (2.25 to 2.84)	0.834	±2.5	0.82^¥^ (0.51 to 0.94)
[La^−^]_Rest_ (mmol·L^−1^)	1.1 ± 0.4 (0.9 to 1.3)	1.2 ± 0.4 (1.0 to 1.5)	0.444	±16.1	0.17 (−0.39 to 0.64)
[La^−^]_Peak_ (mmol·L^−1^)	12.6 ± 1.5 (11.7 to 13.4)	12.8 ± 1.3 (12.0 to 13.9)	0.534	±2.8	0.52 (−0.00 to 0.82)
Δ[La^−^] (mmol·L^−1^)	11.4 ± 1.6 (10.5 to 12.3)	11.6 ± 1.3 (10.8 to 12.3)	0.736	±2.4	0.49 (-0.05 to 0.81)
E_PCr_ (L)	1.53 ± 0.34 (1.33 to 1.73)	1.45 ± 0.42 (1.21 to 1.69)	0.448	-3.4	0.55^*^ (0.02 to 0.82)
A_1_ (mL·kg^−1^·min^−1^)	21.8 ± 4.4 (19.3 to 24.4)	21.1 ± 6.5 (17.3 to 24.8)	0.607	-2.8	0.54^*^ (0.01 to 0.83)
τ_1_ (min)	0.98 ± 0.21 (0.86 to 1.11)	1.03 ± 0.54 (0.72 to 1.34)	0.768	±11.8	−0.17 (−0.64 to 0.39)

*Values are Mean ± SD (CI95%)*.

* *P < 0.05*;

¥ *P < 0.01; E_OXID_ = net aerobic contribution; E_[La]_ = glycolytic metabolism; [La^−^] = blood lactate; E_PCr_ = phosphagen metabolism; A_1_ = amplitude 1; τ_1_ = time constant 1*.

## Discussion

The aim of the study was to verify the effects of acute caffeine supplementation on AC_[La−]+EPOCfast_ in cycling. The main finding of this study was that acute caffeine supplementation improved the time to exhaustion during the supramaximal effort at 115% of i$\overset{{}^{\circ}}{V}O_{2max}$, but the AC_[La−]+EPOCfast_ remained unaltered.

It has been shown that acute caffeine supplementation affects performance and the conventional MAOD estimate (3, 4). Bell et al. (3) found an improvement in the time to exhaustion at 125% of $\overset{{}^{\circ}}{V}O_{2}$ peak intensity and ~7% increase in the MAOD value after supplementation with 5 mg·kg^−1^ of caffeine in untrained subjects. Corroborating these findings, Doherty (4) found an improvement in the MAOD values (~10%) and time to exhaustion (~14%) in untrained subjects with the same caffeine dosage. The explanation of these authors for the improvement in MAOD values was the greater mobilization of the glycolytic metabolism, leading to high production and accumulation of lactate. However, Simmonds et al. (25) found that the iso-time accumulated $\overset{{}^{\circ}}{V}O_{2}$ and oxygen deficit during supramaximal efforts were similar with caffeine (5 mg·kg^−1^) and placebo supplementation. In addition, these authors found no differences in the $\overset{{}^{\circ}}{V}O_{2}$ kinetics during the supramaximal efforts, demonstrating that the greater MAOD values in the caffeine than placebo condition were not related to changes in the relative contribution of the aerobic and anaerobic metabolisms, but were more related to increased time to exhaustion and a consequently greater deficit. In contrast to the results of these authors, in the present study we observed an improvement in time to exhaustion (Table 3), but not in AC_[La−]+EPOCfast_ values after caffeine ingestion.

These differences between the results found in the literature and in the present study can possibly be explained by the methodological issues involved in determining the MAOD and AC_[La−]+EPOCfast_. Medbø et al. (9) suggested that the supramaximal test to determine the MAOD should cause exhaustion in a minimum of 2 min due to the need to achieve the maximum capacity of energy production through anaerobic pathways. These authors demonstrated that MAOD increased with increasing time to exhaustion, reaching a plateau from 2 min on. In both the studies of Bell et al. (3) and Simmonds et al. (25) the mean values of time to exhaustion in the placebo condition (108.2 ± 8.9 s and 93.5 ± 24.1 s, respectively) were lower than the recommended time (i.e., 2–3 min) (26). Thus, this could have caused underestimation of MAOD values in the placebo condition and demonstrated a value closer to the “real” MAOD in the condition supplemented with caffeine due to the increased time to exhaustion. Considering the Simmonds et al. (25) findings that the on kinetics of $\overset{{}^{\circ}}{V}O_{2}$ are not altered with caffeine supplementation, the higher time to exhaustion causes an increase in the oxygen deficit. It is worth emphasizing that the AC_[La−]+EPOCfast_ does not present this “limitation” as it is based on blood lactate and EPOC responses, and not directly on time to exhaustion. In addition, it has been shown that AC_[La−]+EPOCfast_ remains unaltered at different supramaximal intensities (8).

Astorino et al. (1) found that acute supplementation with caffeine changed the magnitude of EPOC after a resistance training session. However, these authors analyzed the area under the EPOC curve, whereas in the present study we analyzed kinetics using a mathematical fitting to derive EPOC_FAST_, which is thought to estimate the phosphagen metabolism pathway (6–8). Regardless of the EPOC method of analysis, the variables from the mathematical adjustment (i.e., A_1_ and τ_1_) used to determine the E_PCr_ in the present study were not statistically different. These findings do not corroborate Poli et al. (2) who found no significant differences in AC_[La−]+EPOCfast_ determined in running after caffeine and placebo supplementation, but reported a lower τ_1_ in the caffeine compared to placebo condition. The main argument of the authors for these differences in τ_1_ is related to the greater exhaustion $\overset{{}^{\circ}}{V}O_{2}$ that allows a faster drop in $\overset{{}^{\circ}}{V}O_{2}$ after the effort. Nevertheless, in both studies the E_PCr_ was not significantly different in the caffeine and placebo conditions. Thus, considering that the effect of caffeine does not seem to be related to increased anaerobic energy supply and a possible effect on E_PCr_ in the caffeine condition would not be “wise,” the E_PCr_ estimated by the EPOC_FAST_ does not seem to be influenced by this ergogenic.

Mechanisms explaining the improvement in time to exhaustion after caffeine supplementation have been inconclusive. Possible explanations could be related to the effects of caffeine on stimulation of the central nervous system, an improvement in neuromuscular transmission, and in the contractility of muscle fiber (4). Simmonds et al. (25) suggested that caffeine can alleviate fatigue by maintaining electrolyte homeostasis at the beginning of the effort, maintaining the extracellular K^+^ concentration and improving the action potential of the membrane, allowing muscle contraction and performance for a longer period before the onset of fatigue. However, in the present study the factors that could explain these mechanisms were not addressed.

However, the ergogenic effect of caffeine on performance is variable and seems to be associated to CYP1A2 polymorphism (27, 28), explaining the variable individuals effects with caffeine ingestion. In a recent study, Guest et al. (27) reported that some individuals are more responsible to caffeine ingestion than other 10 km cycling time-trial performance. The caffeine is metabolized by the CYP1A2 enzyme and these authors found that individual with CC genotype (i.e., homozygous slow metabolizers) decreased the performance compared to individuals with AA (fast metabolizers) and AC (heterozygous slow metabolizers) genotypes after 4 mg/kg intake of caffeine. These authors reported that in 101 participants 49% were AA, 43% AC, and 8% CC, and these range distributions can probably explain the inter-individual variations on performance and mainly on anaerobic capacity in the current study, with inter-individual range for anaerobic capacity change with caffeine compared with placebo was −24.5 to 34.2%. In addition, the Figure 2B shows that 10 of 14 subjects were positive responders to caffeine for performance while 2 were “non-responders” and 2 “negative” responders, evidencing that caffeine ingestion does not affect the anaerobic estimation.

A possible limitation of the study was the absence of$\overset{{}^{\circ}}{\;V}O_{2}\;$kinetics analysis, which could partly explain the improved performance in the supramaximal effort and the unaltered AC_[La−]+EPOCfast_ values. In addition, no analyses were performed related to the effects of caffeine on metabolite accumulation, or intracellular concentrations of K^+^, or any analysis to explain the mechanisms related to the effects of caffeine on the stimulation of the central nervous system, which could contribute to clarifying the issues raised. Finally, it is important to report that the current study did not investigate genetic variation and its potential effects on the relationship between caffeine and the outcomes, and therefore this highlighted the individual responses with caffeine ingestion.

Therefore, we concluded that based on the overall comparison of mean values the acute caffeine supplementation improves the time to exhaustion in a supramaximal effort, although the E_PCr_, E_[La]_, and AC_[La−]+EPOCfast_ remain unaltered. However, the effect of caffeine on the performance and anaerobic capacity depend on the individual.

## Perspective

The AC_[La−]+EPOCfast_ method has been considered an advantageous method due to the reduced time taken to estimate anaerobic capacity (6–8). In addition, this method allows discrimination of the energetic equivalent of both the glycolytic and phosphagen pathways. For this reason, the effects of different ergogenic resources specifically on metabolic pathways have been investigated (16). Caffeine intake increases the time to exhaustion in the supramaximal effort and therefore possibly influences the determination of MAOD by the conventional method (25). However, AC_[La−]+EPOCfast_ does not appear to be time-to-exhaustion dependent (8) and the present study investigated the possible effects of caffeine due to evidence of an impact on lactate concentrations (3) and on EPOC (1). This could influence the AC_[La−]+EPOCfast_ values due to changes in the energy equivalents of the glycolytic and phosphagen pathways. However, the Figures 2, 3 which show individual data strongly suggest that individual responses to the caffeine vary quite markedly. Therefore, the findings of the present study showed that acute caffeine intake increases the time to exhaustion in the supramaximal effort, however does not alter the components of the E_PCr_ (i.e., amplitude and constant time of the mathematical adjustment), E_[La]_ (i.e., lactate concentrations), and consequently the AC_[La−]+EPOCfast_

## Author contributions

WM participated in data acquisition, analyses and writing the manuscript. RB participated in manuscript writing. FN participated in the study design. RdP participated in data acquisition and analyses. AZ participated in the study design and writing the manuscript. All authors read and approved the final manuscript.

### Conflict of interest statement

The authors declare that the research was conducted in the absence of any commercial or financial relationships that could be construed as a potential conflict of interest. The reviewer BG and handling Editor declared their shared affiliation

## Abbreviations

[La^−^]: blood lactate concentration
[La^−^]_Peak_: peak of blood lactate concentration
[La^−^]_Rest_: rest blood lactate concentration
AC_[La−]+EPOCfast_: anaerobic capacity alternative estimation
E_[La−]_: glycolytic pathway
E_OXID_: oxidative metabolism contribution
E_PCr_: phosphagen pathway
EPOC_fast_: fast component of the excessive post-exercise oxygen consumption
GXT: graded exercise test
HR: heart rate
i$\overset{{}^{\circ}}{V}O_{2max}$: intensity associated with maximal oxygen uptake
MAOD: Maximal accumulated oxygen deficit
RER: Respiratory exchange ratio
RPE: ating of perceived exertion
VO_2_: oxygen consumption
$\overset{{}^{\circ}}{V}O_{2max}$: maximal oxygen uptake
SWC: Smallest worthwhile change
